# Effects of Water Provision and Hydration on Cognitive Function among Primary-School Pupils in Zambia: A Randomized Trial

**DOI:** 10.1371/journal.pone.0150071

**Published:** 2016-03-07

**Authors:** Victoria Trinies, Anna N. Chard, Tommy Mateo, Matthew C. Freeman

**Affiliations:** 1 Department of Environmental Health, Rollins School of Environmental Health, Emory University, Atlanta, Georgia, United States of America; 2 FHI 360, Schools Promoting Learning Achievement through Sanitation and Hygiene (SPLASH), Lusaka, Zambia; TNO, NETHERLANDS

## Abstract

There is a well-established link between hydration and improved cognitive performance among adults, with evidence of similar findings among children. No trials have investigated the impact of water provision on cognitive performance among schoolchildren in hot and arid low-resource settings. We conducted a randomized-controlled trial in five schools with limited water access in Chipata district in Eastern province, Zambia, to assess the efficacy of water provision on cognition. Pupils in grades 3–6 were randomly assigned to either receive a bottle of drinking water that they could refill throughout the day (water group, n = 149) or only have access to drinking water that was normally available at the school (control group, n = 143). Hydration was assessed in the morning before provision of water and in the afternoon through urine specific gravity (U_sg_) measured with a portable refractometer. In the afternoon we administered six cognitive tests to assess short-term memory, concentration, visual attention, and visual motor skills. Morning prevalence of dehydration, defined as U_sg_≥1.020, was 42%. Afternoon dehydration increased to 67% among the control arm and dropped to 10% among the intervention arm. We did not find that provision of water or hydration impacted cognitive test scores, although there were suggestive relationships between both water provision and hydration and increased scores on tests measuring visual attention. We identified key improvements to the study design that are warranted to further investigate this relationship.

***Trial Registration*:** ClinicalTrials.gov NCT01924546

## Introduction

There is considerable evidence that dehydration can negatively impact cognitive abilities. Improved access to drinking water in schools may improve children’s ability to learn by improving attention, concentration, and short-term memory. According to UNICEF, only 51% of schools in low-resource settings have access to water throughout the day [1]. Dehydration is likely high in these water-scarce schools, especially during the dry season, although prevalence estimates are unavailable. Evidence on the association between hydration and cognitive performance would provide important data for policy makers in the education sector by establishing a direct link between investments in water at school and educational attainment.

Among adults, exercise-induced dehydration has been shown to impact memory, attention, and visuospatial function [2–7]. In addition to the direct physiological effect of dehydration on cognitive performance, dehydration has also been shown to have a negative effect on psychological factors that could affect cognitive performance, including mood, perceived effort, and concentration [7, 8].

A smaller body of research has explored the cognitive effects of dehydration among children; evidence from intervention and cross-sectional studies in the United Kingdom, Italy, and Israel corroborates findings among adults, suggesting that drinking water is associated with improved attention, short-term memory, visual search, and mood [9–13]. The majority of these studies among children have been conducted in the global North and estimate the effect of voluntary dehydration, which occurs when children do not drink a sufficient amount of water even when it is available [14].

Little research has been conducted in the context of water-scarce environments, where there is the potential for increased prevalence and severity of dehydration due to the fact that children often do not have access to any drinking water throughout the school day. In 2013, we conducted a pilot field trial in Mali to develop testing protocols and field measures of hydration and cognition, as well as to examine the effect of drinking supplementary water during the school day on hydration status and on cognitive performance using a school-level crossover study design. The pilot study revealed challenges with the crossover study design and considerable learning effects related to the novelty of the cognitive tests.

Using the hydration and cognitive testing measures piloted in Mali, the objective of this study was to assess the impact of drinking water provision on cognition among schoolchildren in water-scarce environments. As secondary objectives, we measured the effect of hydration on cognition and the effect of drinking water provision on hydration. Lessons learned from the pilot study have been integrated into the current study. This study was registered at ClinicalTrials.gov NCT01924546.

## Materials and Methods

### Setting

Data were collected over five days in September 2013 in five rural schools in Chipata District, Eastern Province, Zambia. Schools were purposively selected based on accessibility from Chipata town and lack of a water point within 500 meters of the school grounds. Three of the schools independently provided a limited quantity of drinking water for pupils on the day of the study. Maximum ambient outdoor temperature on the day of data collection ranged from 80–95°F (27–35°C) with a mean of 88°F (31°C), and relative humidity ranged from 7–28% with a mean of 17%.

### Sample size calculation

Sample size calculations were based on cognitive test scores from the pilot study in Mali as well as data published by Edmonds & Burford collected using tasks similar to those employed in this study [10]. These data showed a 10–43% increase in test scores after provision of water, and we estimated that between 10 and 184 pupils were needed per study arm, depending on the test (Table 1). We selected the maximum sample size of 300 pupils allowed by logistical and financial considerations.

**Table 1:** Sample size calculations.

Task	Control mean	Control SD	Water provision mean	Water provision SD	Sample size, per arm^a^
*Data from Mali pilot*					
Letter cancellation	18.47	5.48	25.38	5.43	10
Image Difference, direct	1.40	1.28	2.00	1.71	100
Image Difference, indirect	1.52	1.61	2.14	1.71	113
Number recall, forward	5.20	1.41	5.73	1.55	123
Number recall, reverse	3.92	1.47	3.48	1.55	184
Line trace	11.05	7.28	18.83	5.48	11
*Data from Edmonds & Burford [10]*					
Letter Cancellation	29.27	5.90	32.44	4.55	44
Image Difference, direct	1.80	1.06	2.41	0.80	24
Image Difference, indirect	3.83	1.05	4.73	1.49	33

^a^ Sample size calculated using a power of 80% and a two-sided α = 0.05 using difference of means function in OpenEpi [15]

### Participants

**Fig 1:**
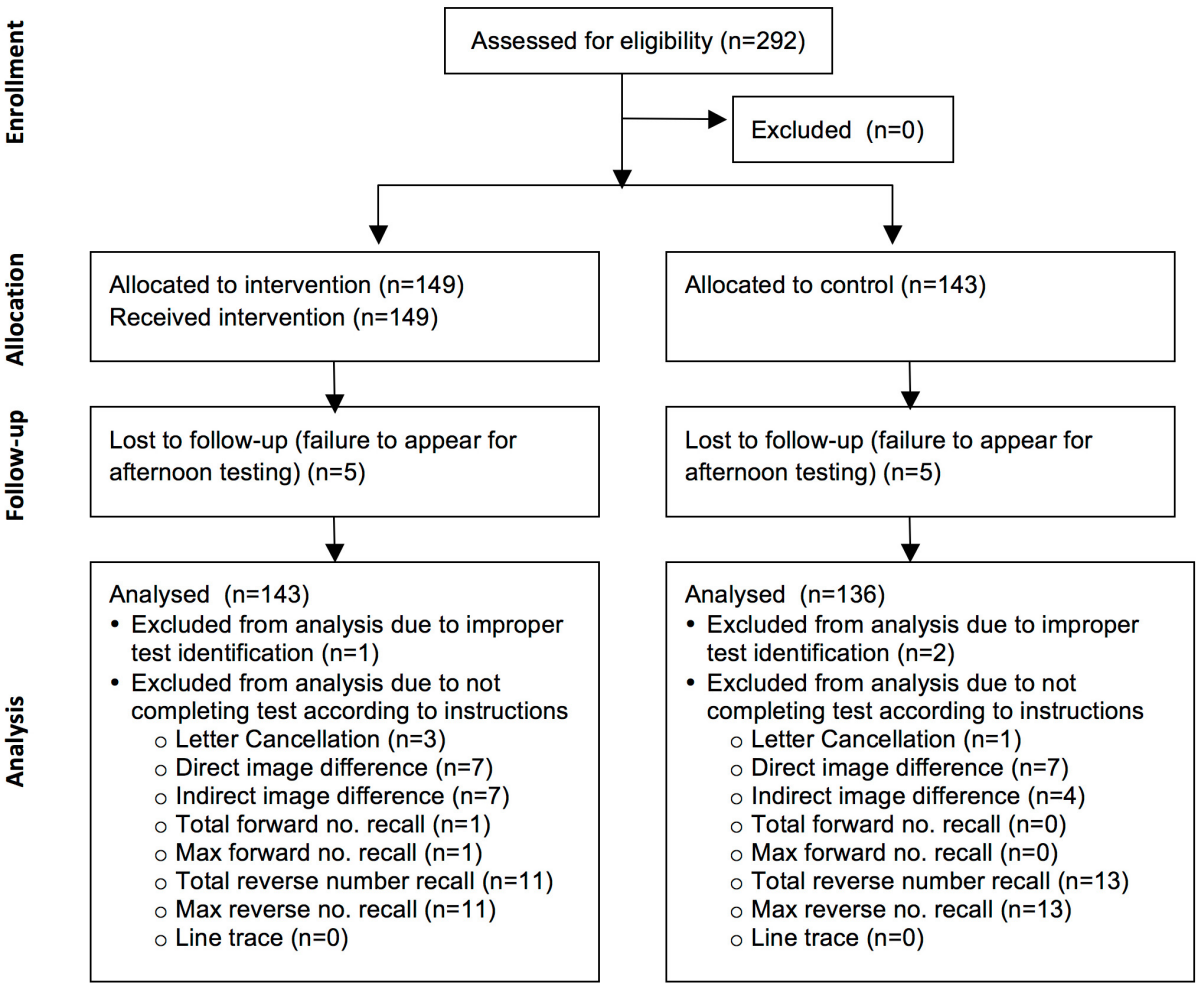
Flow diagram of pupil allocation.

Before data collection activities began at each school, parents of all pupils in grades 3–6 were invited to an informational meeting and asked for consent for their children to participate in the study. All pupils whose parents provided consent were invited to participate on the day of data collection. A total of 292 pupils received parental consent and provided assent for participation (Fig 1).

### Study design

On the morning of each day of data collection, the signed parental consent forms for each school were manually sorted to achieve randomization. Pupils were called in order of sorting and asked for their assent to participate in the study. After providing assent, the pupil was alternately assigned to either the intervention or control arm. All pupils were asked a brief questionnaire concerning their morning eating and drinking habits, mode of transportation and distance to school, and recent illness. Pupils were then given a urine collection container and asked to provide a morning urine sample. After providing the urine sample, pupils in the intervention arm were given a bottle of drinking water and told that they could refill their bottles throughout the day using treated drinking water provided by the study. Pupils in the control arm were not given supplemental water until after the completion of study activities; however, they were permitted to drink any water that was normally available at the school. Between morning data collection and lunch, pupils were given leisure time. We did not record what activities pupils engaged in during that time. After lunch, which was provided at the school, pupils were asked to provide an afternoon urine sample. Study staff then administered a panel of cognitive tests.

### Hydration measures

Hydration was measured through urine color (U_col_) and urine specific gravity (U_sg_), both inexpensive measurement methods that are portable and easy to use in field settings. U_col_ and U_sg_ are highly correlated with urine osmolality (U_osm_), a standard measure of hydration in non-laboratory settings [12, 13, 16–18].

U_col_ was measured using a validated eight-color scale [17]. Two study staff independently assigned each sample a color value between 1 and 8. Differences of ±1 were averaged; if there was a greater difference, a third enumerator was consulted. U_sg_ is a measure of the ratio of solutes in urine compared with water, and normally ranges from 1.003 to 1.030, with a higher number indicating lower hydration [19]. We classified pupils as dehydrated if they had a U_sg_ reading of 1.020 or greater, corresponding to the cutoff for dehydration of U_osm_>800 mOsmol kg^-1^ H_2_O that has been used in previous studies of dehydration among children [12, 13, 18, 20]. U_sg_ was measured using a portable handheld refractometer (Atago model 2791).

Urine samples were collected in the morning between 9am-12pm and in the afternoon between 2–3:30pm. All urine analyses were conducted on the school grounds.

### Cognition measures

Pupils completed six tests measuring four cognitive skills. Tests were taken from research on hydration and cognition among children in Israel and the United Kingdom [9, 10, 13]. The selected tests were based on published test batteries and were designed to test cognitive skills known to be affected by hydration [10, 13]. The tests and instructions were piloted and adapted for use in sub-Saharan Africa during the pilot in Mali. The piloting process included determining whether any of the identified tests were too difficult or too easy for the population, determining which grades the tests were suitable for, and developing appropriate test instructions. We determined that pupils in grades 3–6 were able to complete the tests and a low percentage of children (1–15%) achieved either the maximum or minimum score on any of the cognitive tests, indicating that the selected tests were neither too difficult nor too hard. The tests and instructions were further piloted and adapted in Zambia to ensure that pupils there were equally able to complete the tests. The tests included:

1. Letter cancellation (visual attention): Pupils were given one minute to cross out target letters randomly dispersed among a grid of non-target letters. A score was calculated from the number of correct letters crossed out minus the number of incorrect letters (maximum score 38).
2. Direct image difference (visual attention): Pupils were given one minute to circle differences between two similar images. A score was calculated from the number of circles correctly identifying a difference minus the number of incorrect circles (maximum score 11).
3. Indirect image difference (visual memory): Pupils were given one minute to study an image, then turn to a blank page, then turn to a second, nearly identical image. Pupils were given one minute to circle differences between the second image and their recollection of the first image. A score was calculated from the number of circles correctly identifying a difference minus the number of incorrect circles (maximum score 9).
4. Forward digit recall (short-term memory): Ten sequences of numbers of two to six digits in length were read aloud. Pupils wrote down each sequence after it was read. Two scores were calculated from the total number of correctly recalled sequences (maximum score 10) and the digit span of the longest correctly recalled sequence (maximum score 6).
5. Reverse digit recall (short-term memory): Eight sequences of numbers of two to five digits in length were read aloud. Pupils wrote down each sequence in reverse order after it was read. Two scores were calculated from the total number of correctly recalled sequences (maximum score 8) and the digit span of the longest correctly recalled sequence (maximum score 5).
6. Line trace (visuomotor skills): Pupils were given fifteen seconds to draw a line between two curved parallel lines. A score was calculated from the distance of the drawn line in centimeters minus the number of times the drawn line touched the sides (maximum score 29).

Cognitive tests were conducted immediately following provision of the afternoon urine sample. Total testing time was 60–75 minutes. All tests were paper-based and administered by trained study staff in a group setting in a school classroom. Instruction scripts for each test were translated into Chinyanja and all test instructions included a practice example. Parallel versions of the image comparison tests were created to minimize copying. We piloted several comparable images for the direct and indirect image difference tests, each with the same possible maximum score. The distribution of scores were visually assessed for normality and we selected the four images with the best outcomes for inclusion in the final study. Scores for individual tests were omitted from analysis if responses did not meet criteria for completing the test correctly.

### Data analysis

Data were entered into Microsoft Excel and analyzed using STATA v.13 SE. Independent samples t-tests were used to evaluate differences between study arms in pupil age, grade, distance walked to school, and morning and afternoon U_col_ and U_sg_. Chi-squared tests were used to evaluate differences between study arms for pupil sex, eating and drinking before school, illness in past two weeks, test version, and morning and afternoon dehydration. Paired t-tests were used to evaluate the association between pupils’ individual morning and afternoon hydration measures.

We conducted an intention to treat analysis to evaluate the impact of the intervention (supplementary water provision) on eight cognitive test scores. Secondary analyses included evaluating the association between hydration (U_sg_) and test score, regardless of intervention status; evaluating the association between dehydration (U_sg_≥1.020) and test score, regardless of intervention status; and evaluating the impact of the intervention on hydration (U_sg_) and dehydration (U_sg_≥1.020). Multivariable linear regression models were employed to evaluate the impact of the intervention on each of the eight cognitive test scores, and to test the association between hydration (U_sg_) and test scores, dehydration (U_sg_≥1.020) and test scores, and intervention status and hydration (U_sg_). Logistic regression models were employed to test the impact of the intervention on dehydration (U_sg_>1.020).

Models were assessed for interaction and confounding with sex, age, grade, reported drinking and eating in the morning before school, distance walked to school, illness in the past two weeks, test version, morning hydration, morning dehydration, and the availability of drinking water at the school. Variables were included in the models if they had a p-value less than 0.006, the level of significance determined by using the Bonferroni correction to account for eight hypotheses on a single outcome. Illness in the past two weeks and the availability of drinking water at the school appeared as significant modifiers in the relationship between U_sg_ and some test scores. Test version appeared as a significant confounder for the relationship between study arm and the indirect image difference test, and morning dehydration appeared as a significant confounder for the relationship between both hydration outcomes and the direct image difference test. These confounders were included in the final models as warranted.

### Ethical approval

This study was approved by the Emory University Institutional Review Board (Atlanta, GA), the FHI 360 Office of International Research Ethics (Durham, NC) and the Excellence in Research Ethics and Science Ethical Review Board (Lusaka, Zambia). Written permission to conduct the study was obtained from the Eastern Province Provincial Education Officer. Prior to conducting the study in each school, the study team met with the school management committee and the school head teacher to explain the study, the rights of the pupils, and to obtain permission to conduct the study. Signed parental consent and verbal pupil assent were secured for all study participants.

## Results

Data from 279 pupils were included in the final analysis (see Fig 1). Ten of the pupils who provided assent were lost to follow up due to failing to appear for afternoon testing. Three pupils were excluded from cognitive test analysis due to improper labeling, and three pupils in the control group did not provide afternoon urine samples and were excluded from hydration analyses. Seventy-nine individual cognitive test scores were excluded from analysis due to pupils not completing the test according to directions.

### Demographics

Pupils were in grades 3–6 and their ages ranged from 8–17. The intervention and control samples were comparable in terms of sex, age, grade, eating and drinking habits on the morning of testing, illness in past two weeks, and distance walked to school (Table 2). There was a significant difference in distribution of the two test versions between intervention and control arms due to errors in the allocation of test packets.

**Table 2:** Descriptive statistics of sample population by study arm.

	Control (*N* = 133)	Intervention (*N* = 143)	
	n (%)	n (%)	*p*^b^
Girls	65 (48.9%)	82 (57.3%)	0.16
Age^a^	12.6 (1.7)	12.7 (1.8)	0.88
Grade^a^	4.6 (1.1)	4.6 (1.1)	0.77
Ate before school	47 (35.1%)	64 (45.1%)	0.09
Drank before school	49 (36.8%)	45 (31.5%)	0.35
Minutes to school ^a^	36.7 (29.3)	37.0 (25.0)	0.93
Illness in past 2 weeks	23 (17.2%)	26 (18.2%)	0.82
Test version A	78 (58.2%)	61 (42.7%)	**0.01**

^a^ Results indicate mean (SD)

^*b*^ P-values based on independent samples t-tests for pupil age, grade, and minutes walked to school, and chi-squared tests for pupil sex, eating and drinking before school, illness in past two weeks, and test version.

### Hydration

Overall mean morning U_col_ was 4.06 (SD 1.55) and mean morning U_sg_ was 1.018 (SD 0.006). Using a cutoff of U_sg_≥1.020, 124 (42.8%) pupils were classified as dehydrated in the morning. Results for all morning hydration indicators were comparable between pupils in the intervention and control arms (Table 3).

**Table 3:** Hydration status by study arm.

	Control (*N* = 134)	Intervention (*N* = 143)	
	Mean (SD)	Mean (SD)	*p*^b^
Morning U_col_	4.15 (1.59)	3.95 (1.54)	0.30
Morning U_sg_	1.018 (0.01)	1.018 (0.01)	0.73
Morning dehydration (U_sg_>1.020)^a^	55 (41.0%)	61 (42.7%)	0.79
Afternoon U_col_	4.75 (1.72)	1.90 (1.62)	**<0.01**
Afternoon U_sg_	1.022 (0.01)	1.006 (0.01)	**<0.01**
Afternoon dehydration (U_sg_>1.020)^a^	90 (67.2%)	14 (9.8%)	**<0.01**

^a^ Results indicate n (%)

^b^ P-values based on independent samples t-tests for morning and afternoon U_col_ and U_sg_ and chi-squared tests for morning and afternoon dehydration.

All measures of hydration worsened from the morning to the afternoon for pupils in the control arm. Among the three schools that provided their own drinking water for pupils, the proportion of dehydrated pupils in the control arm in the afternoon was significantly lower than the proportion of dehydrated pupils in the two schools that did not provide any water (77.4% and 59.8%, respectively, p = 0.03). Hydration indicators improved from the morning to the afternoon for pupils in the intervention group.

### Provision of water and cognitive performance

We observed a positive impact of the intervention on scores for the direct image difference test, which measures visual attention (Table 4). We did not observe any clear effect of the intervention on other cognitive tests.

**Table 4:** Mean and standard deviation (SD) of cognitive tests by study arm, with multivariable linear regression models of the association between the provision of drinking water and cognitive performance among pupils in Zambia.

	Control	Intervention	Regression		
Cognitive Test	Mean (SD)	Mean (SD)	Beta^a^	95% CI	*p*
*Visual Attention*					
Letter cancellation (n = 272)	20.4 (7.7)	21.0 (7.5)	0.65	-1.20,2.49	0.49
Image difference, direct (n = 262)	1.9 (1.6)	2.3 (1.5)	0.37	-0.02,0.76	0.05
*Visual memory*					
Image difference, indirect (n = 265)	1.7 (1.9)	1.5 (1.9)	-0.10	-0.55,0.36	0.68
*Short-term memory*					
Number recall total, forward (n = 275)	5.4 (1.2)	5.4 (1.3)	-0.04	-0.35,0.27	0.80
Number recall maximum digit span, forward	4.1 (0.7)	4.1 (0.7)	-0.01	-0.18,0.16	0.91
Number recall, reverse (n = 252)	3.8 (1.4)	3.7 (1.5)	-0.14	-0.51,0.23	0.47
Number recall maximum digit span, reverse	3.3 (0.9)	3.2 (0.9)	-0.10	-0.32,0.13	0.40
*Visuomotor skills*					
Line trace (n = 276)	18.5 (7.4)	19.0 (7.0)	0.56	-1.17,2.30	0.52

^a^ A positive β value indicates an increase in test scores within the water arm. The model for the indirect image difference tests controls for test version.

### Dehydration and cognitive performance

The associations between mean cognitive test scores and dehydration status, regardless of intervention status, are presented in Table 5. We did not observe any clear associations between dehydration status and any of the cognitive test scores. We observed a suggestive association of higher test scores for the letter cancellation test among pupils who were classified as hydrated.

**Table 5:** Mean and standard deviation (SD) of cognitive tests by dehydration status, with multivariable linear regression models of the association between dehydration and cognitive performance among pupils in Zambia.

	Dehydrated^a^	Hydrated	Regression		
Cognitive Test	Mean (SD)	Mean (SD)	Beta^b^	95% CI	*p*
*Visual Attention*					
Letter cancellation (n = 272)	19.6 (6.9)	21.3 (8.0)	1.70	-0.20,3.61	0.08
Image difference, direct (n = 262)	2.0 (1.6)	2.1 (1.6)	0.11	-0.29,0.51	0.59
*Visual memory*					
Image difference, indirect (n = 265)	1.7 (2.0)	1.5 (1.8)	-0.07	-0.54,0.39	0.75
*Short-term memory*					
Number recall total, forward (n = 275)	5.5 (1.1)	5.3 (1.4)	-0.19	-0.51,0.13	0.25
Number recall maximum digit span, forward	4.1 (0.6)	4.1 (0.7)	-0.04	-0.22,0.14	0.68
Number recall, reverse (n = 252)	3.7 (1.4)	3.7 (1.5)	0.04	-0.34,0.43	0.83
Number recall maximum digit span, reverse	3.2 (0.9)	3.2 (0.9)	-0.06	-0.29,0.17	0.62
*Visuomotor skills*					
Line trace (n = 276)	18.6 (7.0)	18.9 (7.4)	0.48	-1.31,2.28	0.60

^a^ Dehydration defined as U_sg_≥1.020

^b^ A positive β value indicates an increase in test scores among hydrated pupils. The model for the direct image difference tests controls for morning dehydration.

### Urine specific gravity and cognitive performance

We found no clear associations between urine specific gravity and cognitive test score, regardless of study arm (Table 6). We saw a suggestive association of higher scores for the letter cancellation test as hydration increased.

**Table 6:** Multivariable linear regression models of the associations between hydration (U_sg_) and cognitive performance among pupils in Zambia.

	Regression		
Cognitive Test	Beta^a^	95% CI	*p*
*Visual Attention*			
Letter cancellation (n = 272)	-7.26	-15.38,0.86	0.08
Image difference, direct (n = 262)	-0.15	-1.86,1.57	0.87
*Visual memory*			
Image difference, indirect (n = 265)	0.94	-1.05,2.94	0.35
*Short-term memory*			
Number recall total, forward (n = 275)	0.14	-1.24,1.51	0.85
Number recall maximum digit span, forward	-0.28	-1.04,0.48	0.47
Number recall, reverse (n = 252)	0.11	-1.54,1.76	0.90
Number recall maximum digit span, reverse	0.38	-0.61,1.38	0.45
*Visuomotor skills*			
Line trace (n = 276)	-3.25	-10.93,4.43	0.41

^a^ A negative β value indicates an increase in test scores as hydration increases. The model for the direct image difference tests controls for morning dehydration.

We observed evidence of effect modification between U_sg_ and certain test scores. We assessed the scores for the direct image difference test among pupils who were ill in the past week (β = 1.53, 95%CI -50.65, 53.71, p = 0.95) and among pupils who were not ill in the past two weeks (β = -14.57, 95%CI -32.83,3.70, p = 0.12) and found no association for either group. We assessed the forward number recall test among pupils in schools where water was available (β = -7.61, 95%CI -18.09,2.87, p = 0.15) and where no water was available (β = 7.45, 95%CI -3.55,18.45, p = 0.18) and found no association.

## Discussion

To our knowledge, this is the first experimental trial to examine the impact of water provision on cognition among children in any low- or middle-income country. We found that a high number of pupils were dehydrated in the morning, dehydration increased throughout the day, and the provision of water significantly decreased dehydration. However, we found little evidence of associations between the provision of water or hydration status on cognitive performance.

The intervention had a significant impact on pupil hydration. We found that 43% of pupils in the study schools were dehydrated in the morning. Unrestricted access to drinking water under the intervention condition reduced the prevalence of dehydration to 10% by the afternoon, while pupils in the control condition had an increase of dehydration prevalence to 67%. The increase in dehydration was greater among pupils in the control group in schools that did not provide any water to pupils (77% dehydrated) compared to pupils in schools where some water was available (60% dehydrated). These results suggest that the provision of even a limited quantity of drinking water helped mitigate levels of dehydration; however, this was not sufficient to prevent an increase in dehydration throughout the school day. We did not measure the quantity of water that pupils in the intervention arm consumed. Further research could be conducted to establish the quantity of water necessary for a pupil to maintain hydration throughout the school day.

The proportion of pupils who were dehydrated in the morning (43%) was lower than what had been previously observed in Israel (84%) [18] and Italy (68%) [12]. The variability in the prevalence of dehydration may be explained by research suggesting that mean urine osmolality ranges widely between individuals and cultures [21]. Thus, although the cutoff value we used to evaluate dehydration among children in Zambia (U_sg_≥1.020) was equivalent to those used in previous studies of dehydration among children in Western settings [12, 13, 18, 20], the value corresponding to physiologic dehydration might be different among the Zambian pupil population. Additional research may be needed to evaluate differences in cross-country and cross-cultural urine osmolality, and appropriate cutoff values for hydration classification.

We found few, if any, associations between the provision of water and improved performance on cognitive tests. There was some evidence that provision of supplementary drinking water was associated with increased visual attention as measured through the direct image difference test. However, the p-value for this test at p = 0.05 did not meet the threshold for significance suggested by using the Bonferroni correction to account for multiple comparisons. Our results stand in contrast to previous research finding significant improvements in memory, visual attention, visual memory, and visual search after supplemental water provision [9–11], as well as research demonstrating significant negative impacts of dehydration on short-term memory [12, 13].

We saw some additional evidence suggesting that pupils who were classified as hydrated (U_sg_<1.020) and pupils with lower overall U_sg_ performed better on tests of visual attention. However, the confidence intervals for these associations crossed zero. These findings should be interpreted with caution, as this was a secondary analysis that pooled observations from both intervention and control arms, and as such did not have an unbiased counterfactual group.

There are a number of possible explanations for the lack of significant associations between water provision and cognitive test scores, and between hydration and cognitive test scores.

Unlike the previous research conducted in the UK, Italy, and Israel, our study did not conduct pre- and post-intervention cognitive testing since we had observed considerable learning curves associated with the cognitive tests that we thought may obscure any changes revealed from improved hydration during our pilot in Mali. Without these baseline measures of cognitive performance, we cannot be sure that the intervention and control arms were comparable in terms of cognitive abilities. Also, our study may not have captured additional potential confounders and effect modifiers. Although we did collect self-reported information about illness in the past two weeks, we did not have any biologic measures of soil-transmitted helminth infection, under-nutrition, or malaria, all of which have been shown to negatively impact cognitive development and performance of children [22–24]. We also did not collect information on physical activity during the day, which could have affected both hydration levels and cognitive performance [25]. However, because pupils were randomized to the intervention and control groups, the effects of these potential confounders would have likely been evenly distributed between the groups.

Additionally, we found evidence of excessive water consumption among some children in the treatment group. In at least two of the study schools, some pupils treated their access to water as a competition to drink as much as possible. Seventy-eight (28%) pupils had an afternoon U_sg_ measurement below 1.003, which is the lower limit for normal hydration [19]. Rogers, Kaint, and Smith [26] found that drinking water decreased cognitive performance among people who had low self-reported thirst. It is possible that excessive water drinking impaired cognitive performance for some pupils. We conducted a sensitivity analysis on all models excluding pupils with U_sg_<1.003 and did not detect any significant results. However, it is possible that any effect of excessive water consumption extended to pupils with U_sg_ above 1.003 as well. We did not include measures of self-reported thirst in this study, as prior research suggests that reported thirst is not correlated with biometric indicators of hydration [27–29]. However, future studies may benefit from including self-reported thirst to account for potential effect modification.

Furthermore, our measurement of hydration may not have been sufficiently sensitive. Our use of U_sg_ was motivated by the cost effectiveness and practicality of handheld refractometers that require only a small urine sample, as well as past research that has shown U_sg_ to be highly correlated with U_osm_ [17]. However, other research has indicated that U_sg_ can lag behind other measures of hydration and may not always be an accurate indicator of current hydration status [30, 31]. Although the low cost and ease of measurement in the field provide compelling support for the use of U_sg_ to measure hydration, additional research may be needed to support the use of this measure in future studies of the effect of hydration on cognition. Additionally, it is possible that providing water over the course of one school day may not be sufficient to reduce the negative impacts of dehydration on the cognition of chronically dehydrated schoolchildren. Longitudinal research examining the sustained provision of supplementary drinking water on cognitive performance is warranted.

Our study may have been underpowered, as we did not achieve our sample size target of 300 pupils. Additionally, sample size calculations were based on data from England and Mali and may not reflect the context-specific effects of the impact of hydration on test scores. When we did observe differences in test scores between hydrated and dehydrated pupils in the Zambia trial, the differences were not as large compared to results from similar tests conducted in Israel and the United Kingdom [9, 10, 13].

Finally, we cannot verify that the cognitive tests we employed accurately tested the cognitive skills they were intended to assess. Due to an error in test allocation, the two version of the image tests were not distributed evenly between study arms, and differences in the difficulty of the two versions may have obscured the relationship between water consumption and the cognitive skills we intended to assess. Additionally, although the cognitive tests selected for this study were taken from published test batteries that had been validated to assess specific cognitive domains, there is some concern that cognitive tests developed in Western settings may not be appropriate in other cultural contexts [32]. Western tests have been successfully adapted to contexts in Sub-Saharan Africa [33, 34], and we piloted all tests to ensure that pupils in Zambia could accurately complete them. We did not, however, conduct an assessment to ensure that the tests did indeed assess the intended cognitive functions among this population. Further research may be needed to ensure that measures of cognitive function are suitably adapted for use among pupils in Zambia and other rural sub-Saharan African contexts.

## Conclusions

We found that providing an unrestricted quantity of water decreased dehydration throughout the school day, and that dehydration significantly increased in the absence of a sufficient quantity of drinking water. We found few associations between water provision or hydration status and cognitive test score, although we did see a suggestive relationship between both water provision and hydration and improved scores on tests of visual attention. While there is considerable evidence that dehydration leads to reduced cognitive test scores, this relationship has not been confirmed through a trial of water provision among schoolchildren in a low-resource field setting. It is possible that no association may truly exist between hydration and cognitive performance; however, our inability to find a clear association between provision of water and cognition may be due to logistical challenges with the study design, power, and measurement of outcomes. Current policies to increase water access may not consider the quantity of water that is available to pupils; the impact of access to a sufficient quantity of drinking water for school children deserves further investigation.

## Supporting Information

S1 File Dataset. (XLS)

S2 File Codebook. (XLSX)

S3 File CONSORT checklist. (DOC)

S4 File Study protocol. (DOC)

S5 File Dataset from Mali pilot. (XLS)

S6 File Codebook from Mali pilot. (XLSX)
