# Supplementary material for: Effects of Water Provision and Hydration on Cognitive Function among Primary-School Pupils in Zambia: A Randomized Trial
Source: PLoS One. 2016 Mar 7;11(3):e0150071. doi: 10.1371/journal.pone.0150071 (PMC4780815; doi:10.1371/journal.pone.0150071)
Supplement: S4 File — (DOC) [file pone.0150071.s004.doc]

**RESEARCH PROTOCOL**

**IRB00065861**

**SHORT TITLE** SPLASH Zambia - Dehydration

**STUDY TITLE** Cognitive effects of drinking water and improving hydration status among schoolchildren in Zambia

**INVESTIGATORS**

Matthew Freeman, PhD, MPH Emory University Principle Investigator

Sarah Porter, MPH Emory University Study Staff

Victoria Trinies, MPH Emory University Study Staff

Kaleb Price Emory University Graduate Student Researcher

Justin Lupele FHI360 Co-Investigator

Tommy Mateo FHI360 Study Staff

**SPONSOR** USAID, sub-award from FHI 360

**DATE** April 3, 2013

CONTENTS

[1 Background 2](#__RefHeading___Toc352772412)

[1.1 Specific Aims 2](#__RefHeading___Toc352772413)

[1.2 Preliminary studies supporting this research 2](#__RefHeading___Toc352772414)

[1.3 Study significance 4](#__RefHeading___Toc352772415)

[2 Design 4](#__RefHeading___Toc352772416)

[2.1 Sample 4](#__RefHeading___Toc352772417)

[2.2 Setting 5](#__RefHeading___Toc352772418)

[2.3 Recruitment 5](#__RefHeading___Toc352772419)

[2.4 Procedures 5](#__RefHeading___Toc352772420)

[2.5 Measures 7](#__RefHeading___Toc352772421)

[2.6 Risks to participation 9](#__RefHeading___Toc352772422)

[2.7 Benefits to subject or future benefits 9](#__RefHeading___Toc352772423)

[2.8 Data analysis 9](#__RefHeading___Toc352772424)

[3 Training 10](#__RefHeading___Toc352772425)

[4 Data management and monitoring 11](#__RefHeading___Toc352772426)

[5 Confidentiality 11](#__RefHeading___Toc352772427)

[5.1 Plans to protect privacy of subjects and confidentiality of data 11](#__RefHeading___Toc352772428)

[5.2 Plans to link data to identifiers 11](#__RefHeading___Toc352772429)

[6 Informed Consent 11](#__RefHeading___Toc352772430)

[6.1 Written consent from school and government 11](#__RefHeading___Toc352772431)

[6.2 Pupil assent 12](#__RefHeading___Toc352772432)

[7 Sharing of findings 12](#__RefHeading___Toc352772433)

[8 References 12](#__RefHeading___Toc352772434)

# Background

## Specific Aims

The purpose of this study is to evaluate the impact of improved water access on the cognitive performance and mood of primary school children in sub-Saharan Africa. Emory researchers have recently quantified the impact of improved water, sanitation, and hygiene (WASH) access in school on health and school attendance. However, the specific impact of access to drinking water during the school day on cognitive abilities linked to educational attainment has never been studied in low-resource and arid settings, where UNICEF estimates that only 47% of schools have access to any water during the day.[^6^](#_ENREF_6)

There is a considerable body of research assessing links between dehydration to reduction in cognitive ability and mood, although few of these studies involve children and only one such study has taken place outside of Europe.[^10^](#_ENREF_10) Previous studies of cognitive functions including memory and attention have linked them to necessary educational skills in reading and arithmetic.[^11^](#_ENREF_11) This study therefore presents the opportunity to document the direct impact of improved school WASH on cognitive processes that influence educational outcomes. A better understanding of the impact of water intake on cognition among pupils in low-income settings has importance for the growing number of school-based WASH interventions in developing countries, particularly for advocacy from donors and national governments.

To this purpose, we have identified the following objectives:

1. Refine a battery of cognitive tests measuring visual attention, visual memory, short-term memory, and visuomotor skills for children in low-income settings and measure the practice effect of repeated cognitive testing;
2. Assess the levels of dehydration among children in poor and arid settings;
3. Quantify the effect of improved water access on hydration status and cognition; and
4. Compare different field hydration assessment techniques for accuracy and ease of use.

Through our proposed research, we will build upon findings from our recent pilot research in Mali to investigate further the link between hydration and cognition in the Zambian setting.

## Preliminary studies supporting this research

### Cognitive impacts of experimentally-induced dehydration in adults

In humans, a normal state of hydration, or *euhydration*, is closely regulated through a complex system of physiological mechanisms and is achieved through a balance of water intake and loss throughout the day; *dehydration* is defined as a deficit in body water.[^12^](#_ENREF_12)

Numerous studies have indicated an association between dehydration and cognitive function in adults through controlled experiments in which specific levels of dehydration were brought about through either exercise or heat stress. In these experiments, dehydration has been significantly associated with lowered performance on many tests of cognitive ability, including short-term memory, long-term memory, arithmetic efficiency, visuospatial function, and attention. These negative impacts are generally observed only once a moderate level of dehydration was achieved, corresponding to a 2% loss of body mass or greater. Increasing amounts of body water loss beyond this 2% point results in further decline of cognitive abilities. These findings were repeated in studies that induced moderate dehydration through passive water restriction or a more mild type of exercise. These observed cognitive deficits were not dependent on the method used to induce dehydration (heat stress vs. exercise), implying that it is the state of hydration itself and not the stress of either experimental condition that reduces cognitive ability.[^17^](#_ENREF_17)

In addition to cognitive measures, experimentally induced dehydration has been associated with subjective measures of increased of fatigue, loss of ability to concentrate and increased perceived effort for completing tasks.[^18^](#_ENREF_18) More recently, dehydration has been linked to changes in mood in young adult male and female populations. In males, trials involving a 1% or greater body mass loss were associated with an increase in measured tension and fatigue.[^21^](#_ENREF_21) In females, mild dehydration was also associated with adverse changes of mood states of vigor and fatigue, even in cases where cognitive performance was not affected.[^19^](#_ENREF_19) These studies suggest that mood may more sensitive than cognition to lower levels of dehydration in adults.

There are multiple physiological mechanisms that could be responsible for the link between cognitive dysfunction and dehydration.[^16^](#_ENREF_16) One noteworthy brain imaging study in young adults (median age 16.1 yrs) found changes in neuronal activity patterns changes during dehydrated states. While there were no observable changes in cognitive performance, brain function was less efficient and more resources were required to achieve the same effect. Researchers concluded that this extra effort would adversely affect select cognitive functions in states of prolonged dehydration.[^22^](#_ENREF_22)

### Cognitive impacts of water intake in adults

A limited number of studies have assessed the impact of *improving* hydration status, and those that have first purposefully dehydrated individuals under experimental conditions. In one study of fluid intake following dehydration, subjects ingested sufficient fluid to return to their original body mass during a three-hour period recovery period following exposure to dehydrating conditions. While cognitive test performance did not improve in control conditions, fluid intake was observed to mitigate decline in performance on a long-term memory recall task and was associated with a decrease in feelings of fatigue.[^17^](#_ENREF_17) In an experiment where subject hydration status was classified according to self-reported thirst, there was an improvement in performance on tests of sustained attention among “thirsty” subjects following ingestion of water. Subjective measures of alertness among all subjects improved after drinking.[^24^](#_ENREF_24) In a similar study, water intake did not affect cognitive performance but did impact reported alertness.[^25^](#_ENREF_25)

These studies may offer a more relevant example of the impact of water intake compared with induced dehydration studies, in which subjects begin in a state euhydration and are dehydrated temporarily. They indicate that detriments to cognition and changes in mood are not necessarily mitigated by fluid ingestion immediately following dehydration. We are not aware of any research that specifically addresses changes in cognitive function following more prolonged and complete rehydration periods, and this could be an area for further exploration.

### Cognitive impacts of dehydration and water intake in children

The relationship between water access, hydration, and cognition in children remains largely unstudied to date in spite of the fact that children are at greater risk for dehydration than adults.[^26^](#_ENREF_26) Only one study has assessed dehydration prevalence in hot, arid regions. In the Israeli desert, 67.5% of children were observed to be in a state of moderate to severe dehydration.[^27^](#_ENREF_27) This limited evidence available suggests that children residing in hot, arid climates may have a higher-than-normal prevalence of dehydration.

The body of research assessing the relationship between dehydration and cognition in children is not nearly as comprehensive as that of adults; due to ethical constraints, researchers cannot carry out the same controlled experiments in which subjects are purposefully dehydrated in a laboratory setting. Instead, research in this area uses either observational study designs in which associations between voluntary dehydration and cognitive deficits are measured,[^10^](#_ENREF_10) or intervention designs in which cognitive performance is compared between scenarios when additional water is and is not provided.

Data suggest that the cognitive effect of dehydration in child populations is similar as that in adults – a study from Israel found dehydrated children’s short-term memory worsened throughout the day compared to those who were not dehydrated.[^10^](#_ENREF_10) Evidence from three intervention studies in the U.K. and one in Italy is consistent with these findings: drinking water was associated with better scores of attention, short-term memory,[^29^](#_ENREF_29)^, 32^ and visual search.[^30^](#_ENREF_30) In addition, children who voluntarily drank additional water had higher self-reported ratings of happiness, regardless of test performance improvement.[^30^](#_ENREF_30) A limitation to three of these intervention studies is that hydration status was assessed through self-reported thirst only without using additional biometric data.

In March 2013, we completed work in Mali investigating the link between hydration and cognition. Data from this study have not yet been published, but we have completed preliminary analyses. In this study, we saw some evidence of the link between drinking water and cognition; however, practice effect (improvement due to repeated testing) was much stronger than anticipated in the study population, and may have masked the effect of hydration on test scores. We have addressed this challenge in the protocol below to ensure the ability to detect a true difference in test scores.

### Field measures of hydration

While there is no agreed upon “gold standard” for assessing hydration, plasma osmolality is often considered the best available measure.^7^ Laboratory testing for plasma osmolality, however, is expensive and not feasible for use in field settings. Other methods such as self-reported thirst or urine color are often used instead. A limitation of the previous cognitive performance studies in children is their reliance on thirst as a measure of dehydration.^8^

We will use urine specific gravity as our primary biometric measure of hydration. Specific gravity refers to the density of a fluid in relation to pure water, and urine with a density value similar to water indicates better hydration.[^31^](#_ENREF_31) Urine specific gravity is easily measured in field using a drop of urine in a hand-held refractometer and has been validated as an objective measure of hydration status.[^32^](#_ENREF_32)

## Study significance

Data collected through this research will build upon our recently conducted work in Mali. We have allowed for an extended period of pilot testing of cognitive tests and procedures, including formal assessment of practice effect so we can take steps to control for it in our trial. Further refinement of tools and procedures is necessary before they can be implemented in the context of Zambia. We have also modified the study design from a crossover design to a randomized trial. This study will represent the first randomized trial of the impact of water consumption on cognition in sub-Saharan Africa.

# Design

This study is a randomized trial with pre/post testing that will compare change in scores on cognitive tests between two groups. A secondary analysis will be conducted comparing change in test scores of hydrated children and dehydrated children, regardless of experimental group allocation.

We also conduct two pilot assessments of dehydration and cognition independently for the purpose of refining study tools and methods prior to the trial.

This protocol will be submitted to Emory University’s Institutional Review Board (IRB) for approval.

## Sample

### Population

- Total recruitment for all activities will total up to 420 children in up to eight schools.
  - The trial will involve up to 300 children in up to four schools.
  - Hydration piloting activities will involve up to 40 children in up to two schools.
  - Cognition piloting activities will involve up to 40 children in up to two schools.
- Children in grades 3-6 attending primary schools in the SPLASH catchment area in the Eastern Province of Zambia will be eligible. We may limit the population to older grades if we find during piloting that the tests cannot be understood by children in younger grades.

### Vulnerable population safeguards

Prior to data collection, program partner staff will obtain written consent to conduct the research activities in the pupil populations from the appropriate education government offices and from the head of school (using an *en loco parentis*).

Study staff will be trained in ethical considerations related to obtaining informed consent, with special attention paid to obtaining assent from children and avoiding coercion. All pupils will provide assent to participate in the study. We do not anticipate any harmful effects to pupils from participating in any part of this study.

In the trial, all pupils in an eligible grade level will be allowed to participate in cognitive testing activities. If a pupil is not eligible, s/he will be permitted to participate in activities to avoid excluding certain pupils.

### Inclusion criteria

Criteria below applies to trial and pilot activities unless otherwise noted

- School inclusion criteria
  - SPLASH program school
  - No water point within 0.5 km of the school grounds (trial and hydration pilot only)
- Pupil inclusion criteria
  - Grades 3-6
  - Understand and respond to verbal instruction
  - Able to write numbers that have verbally been dictated

## Setting

We will conduct this research with FHI 360, which is implementing the “Schools Promoting Learning Achievement through Sanitation and Hygiene” (SPLASH) program in Zambia. SPLASH is a five-year program funded by USAID taking place in 641 schools in the Eastern Province and Northwestern province. The goal of SPLASH is to improve WASH access in schools.

Data collection for all study activities will take place on the school property.

## Recruitment

The same recruitment process will be used for the pilot activities and the trial.

Potential schools meeting the criteria will be identified by SPLASH. Schools will be contacted by SPLASH to ascertain their interest in participating, will be purposively selected with the partner based on logistical considerations and inclusion criteria. Consent to carry out the study will be provided by the head of school.

All pupils in grades 3-6 will be eligible to participate, regardless of age or sex. Prior to data collection, trained study enumerators will visit the classrooms of eligible grade levels in the study school and explain study procedures. Pupils will be given the opportunity to assent or nor assent to participate privately outside of the classroom following the explanation. Any pupil that does not wish to provide assent will be permitted to be excused from the classroom during testing. Screening for eligibility will take place at the beginning of the pupil survey immediately following the assent procedure. Pupils will be asked to write a string of two numbers that are read to them verbally. Pupils who are unable to complete the task correctly will be ineligible for the study. Pupils that do not meet inclusion criteria will still be permitted to take the tests with their peers, but their results will not be recorded.

## Procedures

The pilot and trial activities will follow different procedures, which are described in detail in this section. **Table 1** summarizes procedures for all study activities.

| **Table 1. Procedures and sample size for study activities** | | | | | | |
| --- | --- | --- | --- | --- | --- | --- |
| **Activity** | **Study design** | **Data collection** | **Intervention** | **# schools** | **Max**  **pupils / school** | **Max**  **sample size** |
| **I. Pilot (cognition)** | Observation | Paper tests | None | 2 | 20 | 40 pupils |
| **II. Pilot (hydration)** | Experiment | Urine sample | Water | 2 | 20 | 40 pupils |
| **III. Trial** | Experiment | Paper tests Urine sample  Interview | Water  (randomized) | 4 | 150 | 300 pupils |
| **TOTAL** | **-** | **-** | **-** | **8 schools** | **-** | **420 pupils** |

### Study design and procedures

1. *Pilot (cognition)*

The purpose of this pilot is to refine the cognitive tests developed elsewhere for the context of Zambia. We will collect observational data on changes in cognitive test performance over a period of five days. This pilot phase will involve only cognition measures. We will conduct activities at up to 2 schools with up to 20 pupils per school. In each of the 2 pilot schools, a maximum of 5 children will be selected from each grade 3-6, totaling no more than 20 children. No water will be provided during this phase of the study. Study staff will lead selected pupils through each task of the paper based cognitive test in a group setting. At the end of each task, staff will discuss with students as a group whether they understood the instructions of each task and how difficult they perceived the task to be. Feedback will be used to refine the test content and instructions before the field trial.

We will repeat testing sessions over five days in order to measure practice effect resulting from repeated testing.

- Time burden testing and feedback is estimated to be a maximum of 90 minutes the first day.
- Testing burden on subsequent days is estimated to take 45 minutes

Cognitive tests are described further in **Section 2.5**, below.

1. *Pilot (hydration)*

The purpose of this pilot of hydration is to assess the effect of water on hydration status. We will conduct an experiment assessing how consuming specific quantities of water affects measured hydration over the course of the school day. The amount of water any individual pupil consumes will be determined by their personal preference and is not intended to achieve a pre-determined level of hydration. We will track the amount of water consumed by weight and compare these against hydration status using urine specific gravity and self-reported thirst. We will conduct activities at up to two schools with up to 20 pupils per school. In each of the two pilot schools, a maximum of five children will be selected from each grade 3-6, totaling no more than 20 children. Pupils will be randomly selected from the school roster.

There will be two arms in this trial: an **intervention arm** and a **control arm**.

- Pupils in the **intervention arm** will receive a bottle of water in the morning and will be eligible to receive refills throughout the day. At every refill, the amount of water will be weighed. At the end of the day pupils will receive a non-consumable prize, such as a small toy or a sticker.
- Pupils in the **control arm** will receive a non-consumable prize in the morning, such as a small toy or a sticker. After data collection has finished, pupils in this arm will receive a full bottle of water to take home.
- Pupils will be instructed not to share water during the day and will be monitored by study staff and teachers to discourage sharing.

Both arms will provide a baseline urine sample and report on thirst before the intervention. At two-hour intervals during the day, pupils will be asked to provide additional urine samples and report on thirst to monitor changes in hydration over time. Over the course of the day, pupils will provide a maximum of four urine samples and report on thirst a maximum of four times.

- Time burden for the urine tests is five minutes per sample.

Pupils will be discouraged from sharing water with others. Pupils will be monitored in the classroom to identify ways to prevent water sharing between students in the intervention and control groups.

Urine sample collection is described further in **Section 2.5**, below.

1. *Randomized trial*

The randomized trial is intended to document the direct link between hydration status and specific cognitive abilities that are linked with educational attainment. The trial will involve two stages:

**Pre-trial.** We will conduct up to five cognitive pre-trial sessions, conducted once per day, to minimize test score improvement due to practice during the trial. The number of pre-trial sessions will be informed by the pilot activities. Pre-trial sessions will orient pupils to the type of testing we are doing and the testing instructions. We will also conduct one short pre-trial interview. We will obtain school informed consent and pupil assent prior to any study activities.

As part of the pre-trial sessions, pupils will also be given a short interview on a day prior to the trial. It will contain questions on drinking and eating habits at school and at home. Pupils will be excused from class to complete the interview one at a time.

**Trial.** Data collection for the intervention trial will take place during one day. On the day of the trial, we will conduct two cognitive testing sessions and collect two urine samples. We will also conduct a short interview asking pupils what they had to eat and drink that morning. Procedures for both arms will be the same, with the exception of the study intervention.

Pupils will be randomly allocated in equal numbers to either the intervention arm or the control arm. We will stratify by gender to ensure an equal proportion of boys and girls in each arm.

- Pupils in the **intervention arm** will receive a bottle of water in the morning and will be eligible to receive refills throughout the day. Pupils will be encouraged to drink throughout the day as they are thirsty but are not expected to consume a specific amount of water. At the end of the day pupils will receive a non-consumable prize, such as a small toy or a sticker.
- Pupils in the **control arm** will receive a non-consumable prize in the morning, such as a small toy or a sticker. After data collection has finished, pupils in this arm will receive a full bottle of water to take home.
- Pupils will be instructed not to share water during the day and will be monitored by study staff and teachers to discourage sharing.

Data collection will follow the schedule below. Small modifications may be necessary depending on the school schedule at the study site.

8:00 – 8:45 Urine sample collection and short interview (Individual).

8:45 – 9:30 Cognitive testing (Group)

9:30 Pupils receive water (intervention arm) or prize (control arm)

3:00 – 3:30 Urine sample collection (Individual)

3:30 –4:15 Cognitive testing (Group)

4:15 Pupils receive water (control arm) or prize (intervention arm)

The pupil interview that will be conducted on a day prior to the trial will contain questions on drinking and eating habits at school and at home. On the trial day, pupils will be asked what they ate and drank at home that morning. Pupils will be excused from class to complete the interview one at a time.

- The time burden for the longer pre-trial interview is five minutes. The time burden for the short interview is negligible.

Pupils will be asked to provide two urine samples on the day of the trial. Pupils will be provided with a urine sample container marked with a unique ID, and given instructions on how to collect the sample. They will return the sample to a study staff member, who will assess hydration on site and then dispose of the sample. If there is no latrine available on site, a private location where pupils provide a sample will be set up. Pupils will always be allowed to provide the sample alone. Study staff will provide water and soap for handwashing so pupils can wash their hands after giving the urine sample.

- Time burden for the urine tests is five minutes per sample.

Cognitive tests will be paper-based and will be administered in a group setting in the classroom by trained study enumerators reading from a standardized instruction booklet. Schoolteachers and administrators will be asked to leave the classroom during the testing period. Each pupil will be provided with a booklet containing a set of six tasks and one scale assessing thirst. If a pupil does not wish to participate in the class activity, s/he will be permitted to leave the class and will be supervised by the teacher. During pre-test sessions, we will make every attempt to minimize time lost from class by conducting sessions during periods where classroom instruction does not take place.

- Time burden for cognitive tests is 45 minutes per session.

## Measures

Measures for pilot and trial study phases will be the same. The pilot phases will be used to refine measures for the trial.

***Hydration status***

All urine measures will be conducted on the school grounds. Study staff will record measurements and then discard the sample. Hydration status will be measured through urine specific gravity, urine color, and self-reported thirst. We may choose to eliminate one or more of these measures from the trial based on findings in pilot activities. The following measurements will be made to assess hydration:

**Urine specific gravity.** Two portable analog refractometers will be used to measure specific gravity of the urine. Two study staff members will read the refractometers. If the readings do not concur within an error .001, the refractometers will be recalibrated and an additional reading will be taken to verify the measurement.

**Urine color.** We will assess urine color as a secondary biometric hydration measurement. We will assign a score to each sample using an 8-point urine color rating scale that has been validated for hydration measurement. Two study staff members will read the chart and the two scores will be averaged. If the readings do not concur within an error of 1 point, a third reading will be taken to verify the measurement.

**Self-reported thirst.** Pupils will be given a scale consisting of a single line with a pictorial description of “not thirsty” on the left side and “thirsty” on the right side. Pupils will be asked to mark on the line where they are in terms of thirst, and ratio of the distance of the mark to the length of the line will serve as the measure of reported thirst.

### Cognition

Cognitive performance will be assessed through a series of six simple tests assessing a range of cognitive skills. The tests have been taken from past research among children in Israel and the United Kingdom, and all of the tests are commonly used measures of cognitive abilities.^6, 30, 31^ All cognitive assessments have been previously piloted in Mali and found to be appropriate for a rural African context. They will be piloted in Zambia prior to data collection and further adapted if necessary for the Zambian context. Local enumerators will be trained on the administration of these tests and we will collaborate with implementing partners to translate test instructions into local languages as needed. All tests will be paper-based tests and will be scored manually.

**Letter cancellation task.** This test assesses *visual attention.* Pupils will be given a grid containing target letters (U) randomly dispersed among non-target letters (O and C). Pupils are given a fixed amount of time to draw a line through as many target letters as possible. Scores are based upon the number of correctly identified targets minus the number of errors.

**Image difference, direct.** This test assesses *visual attention.*[**^30^**](#_ENREF_30) Two nearly identical pictures will be simultaneously presented. Pupils will find the differences between the two. Scores are based upon the number of correctly identified differences minus the number of incorrectly identified differences.

**Image difference, indirect.** This test assesses *visual memory.*[**^29^**](#_ENREF_29) Two nearly identical pictures will be presented, but in sequence. Pupils will be given time to study the first picture, then shown a blank page, then shown the second picture. Pupils will find the differences between the two by circling the differences on the second picture. Scores are based upon the number of correctly identified differences minus the number of incorrectly identified differences.

**Number recall, forward.** This test assesses *short-term memory.*[**^10^**](#_ENREF_10) Sequences of numbers two to eight digits in length will be read aloud, and pupils will immediately recall and record them following the reading. Scores are based upon number of correctly recalled sequences.

**Number recall, reverse.** This test assesses *short-term memory.*[**^10^**](#_ENREF_10) Sequences of numbers two to seven digits in length will be read aloud, and pupils will immediately recall and record them in the reverse direction following the reading. Scores are based upon number of correctly recalled reverse sequences.

**Line tracking task.** This test assesses *visuomotor skills.*[**^29^**](#_ENREF_29)**,** [**^30^**](#_ENREF_30) Pupils will draw a line between two curving parallel lines as quickly as possible while attempting not to touch either of the two lines on the outside within a fixed amount of time. Scores are based upon the number of errors drawing outside the parallel lines and degree of completion of the task, with more errors resulting in lower scores.

**Thirst.** Pupils will provide a self-report of thirst. Pupils will be given a scale consisting of a single line with a pictorial description of “not thirsty” on the left side and “thirsty” on the right side. Pupils will be asked to mark on the line where they are in terms of thirst, and ratio of the distance of the mark to the length of the line will serve as the measure of reported thirst.

## Risks to participation

There are minimal risks to participation in this study. The biggest risk of participation is lost class time. All activities have been designed to minimize time taken away from class instruction, and all efforts will be made not to cause other disruptions. Study staff will ensure that head teachers, other teachers, and pupil participants all fully understand that performance on cognitive tests does not affect their grade, and that urine samples will be discarded.

Response booklets will be stored in a locked file cabinet, and data will be entered into a secure database. No personal identifying information will be kept with the data.

## Benefits to subject or future benefits

The only direct benefit of participation in this study is that all participants will be provided with drinking water during one school day. There are no other direct benefits to participants other than knowing that information gained from this study will contribute to knowledge about the educational and health impacts of improved WASH in schools. The information will be shared with local stakeholders for potential use as an advocacy tool so that future programs in this and other areas can build upon the work that is done in this program.

## Data analysis

### Rationale for proposed number of subjects for dehydration trial

Data published by Edmonds & Burford (2009) collected using tasks similar to those proposed in this study was used to inform the sample size calculations (see table below). Open-Epi v 2.3.1 was used to determine sample size.3

| **Table 2. Sample size calculation** | | | | | |
| --- | --- | --- | --- | --- | --- |
| Task | Group 1 mean | Group 1  SD | Group 2  mean | Group 2  SD | Sample # needed, per arm |
| Letter Cancellation | 29.27 | 5.9 | 32.44 | 4.55 | 44 |
| Identify Differences (direct) | 1.8 | 1.06 | 2.41 | 0.8 | 24 |
| Identify Differences (indirect) | 3.83 | 1.05 | 4.73 | 1.49 | 33 |
| Line tracing | 30.07 | 6.99 | 31.57 | 7.37 | 360 |

We based our sample size on the largest number of pupils needed to identify a difference between groups, within what was logistically feasible for our study. We expect a small number of pupils to refuse participation, and we expect that some pupils allocated to the study group visited on the second day may be lost to follow-up. In addition, while we will equally allocate pupils into a water supplementation group and a control group, one component of our analysis is an observational comparison that classifies pupils by hydration status. Observed prevalence of mild-to-severe dehydration at baseline in hot, arid regions ranged from 53% - 84% in the studies cited in this protocol, so in order to attain 44 pupils in the observed hydrated group, we would need between 77-275 pupils recruited for the study.

We intend to recruit all eligible pupils who wish to participate from grades 3-6 in up to four schools, with a maximum of 150 pupils per school, and a maximum of 300 pupils total from all schools. All pupils within in the selected grade levels will be eligible for recruitment so as not to exclude any pupils from participating.

### Plans for data management and statistical analysis

Results from surveys, cognitive tests, and urine samples will be entered into a password-protected excel database. The database will be exported as the appropriate statistical analysis software file. Paired t-tests will be conducted to compare change in test score from baseline and follow-up between different groups (e.g., water vs. no water; dehydrated vs. hydrated).

### Inclusion of stopping rules as appropriate

Data will be collected between May and August 2013. There are no stopping rules for this study.

# Training

An experienced public health professional will conduct a four-day training with enumerators that includes research ethics, rights and protection of research participants, informed consent process, and data collection tools and procedures. The training will be conducted in English.

Online CITI certification is not feasible in Zambia. Computer literacy is not commonplace, and computers with functioning web access will not likely be available to all study teams. In lieu of CITI training, we will conduct our own ethical training that covers the same topics, including the following themes, with examples given for each:

1. History and purpose of protections for research participants
2. Rights of a research participant:
   1. **Right to** **know** what their participation entails, risks or benefits they’ll receive, level of privacy, what will be done with information they give
   2. **Right to choose** whether to participate, completely free from coercion
   3. **Right to privacy** of the responses they give
   4. **Right to have no harm done** to them as a result of participating or not participating
3. Procedures for obtaining informed assent and assent script
4. Procedures for assessment and response to elevated risk to participants throughout study
5. Procedures for keeping data confidential

At the conclusion of this training, we will administer a comprehension quiz that all enumerators must pass.

See “Ethical training for study staff.doc” under miscellaneous documents for full training outline.

# Data management and monitoring

This trial does not entail a medical intervention of any kind; therefore, a typical DSMP is not necessary. Study staff will be instructed to monitor any unforeseen situations that suggest an increase in risk to participants and immediately inform the study manager so that a decision can be made on how to mitigate those risks.

# Confidentiality

## Plans to protect privacy of subjects and confidentiality of data

Pupils will be assigned a unique identifier at the start of data collection. Stickers with this identifier will be placed on all pupil testing materials. A list linking identifiers to pupils will be created to assist data collection logistics during the course of the data collection period; however, this list will be disposed of after the data collection period is ended. No personal identifying information will be collected as part of this study.

## Plans to link data to identifiers

No personal identifiers will be kept in the database. All samples will be destroyed before leaving the study site and no samples will be banked as part of this study.

# Informed Consent

## Written consent from school and government

A waiver of parental consent for pupil data collection will be secured from the government authority body such as the Ministry of Education. In the local setting, a signed consent will also be obtained from the school director. The director will be asked to sign *in loco parentis* (“in the place of parent”) on behalf of the pupil participants. The consent procedure will inform the school of the activities, purpose of the research, and will obtain permission for the researchers to carry out activities with assenting pupils.

We are requesting that parental consent be waived under [45 CFR 46.116(d)](http://www.hhs.gov/ohrp/humansubjects/guidance/45cfr46.html#46.116). First, we believe that the research involves no more than minimal risk to the pupils participating, since their participation is limited to minimal loss of class time and the provision of urine samples. Secondly, we believe that the research will not adversely affect their rights or welfare, particularly given that we will not conduct the research unless given permission by the school directors that have been entrusted with their care. Third, given the number of students that will be participating in the study, securing permission from each parent resents impracticable logistic challenges. Having enumerators travel to the homes of pupils would require excessive time and cost due to a widely dispersed population and the difficulty of locating parents who work outside the home. Calling parents to the school would risk lost wages, and sending a note home with the child for parental consent is not feasible given low literacy rates in rural Zambia. Finally, school management would be given informational materials and encouraged to share information with parents regarding the details of the study at any time.

For these reasons, we are seeking a waiver of parental consent from an authority body. In the place of parents, we are seeking the permission of the school directors and/or school management committees, who will grant permission representing students’ and parents' interests.

School directors and/or school management committee chairs would sign the *in loco parentis* form for up to 150 pupils at each school for the trial (see “TRIAL Loco Parentis consent.doc”) and up to 20 pupils at each school for the pilot (see “LEARNING Loco Parentis consent.doc” and “HYDRATION Loco Parentis consent.doc”)(see Table 1). This form will be signed at the first visit, and will serve as consent for all subsequent visits. One *in loco parentis* form will be collected from each school, with up to eight total being collected for this study (one for each of the schools).

## Pupil assent

All pupil subjects in the classes selected to participate in the study will be read a complete informed oral assent script as a group prior to any data collection by a trained staff member. The staff member will then call the pupils outside of the class one by one and ask if they have any questions about the explanation that was given in class, and answer any questions they may have. They will then ask if the pupil assents, and record assent on the list of pupils. While the study will be explained to the class as a group, the actual pupil assent will be taken in this private setting so that there is no appearance of coercion from classmates or teachers.

Once a pupil assents, the study staff member will commence with the screening test. If the pupil passes the test, the study staff will administer the pupil questionnaire. Once the questionnaire is complete, the pupil will be given a collection cup and asked to provide a sample.

Oral assent has been chosen rather than written due to varying levels of literacy at the school as well as a desire to minimize paperwork that contains participants’ names.

A maximum of 150 assents will be recorded at each study school for the trial (see “TRIAL Student Assent Script.doc”), and 20 at each school for the pilot activities (see “LEARNING Student Assent Script.doc” and “HYDRATION Student Assent Script.doc”), with a maximum of 420 total pupil assents recorded over the course of the study (see Table 1).

# Sharing of findings

A report highlighting key points from the research findings will be given to study partners once data has been analyzed.

# References

1. Freeman M, Clasen T, Brooker S, Akoko D, Rheingans R, 2011. The impact of a school-based hygiene, water treatment, and sanitation intervention on reinfection with soil transmitted helminths in western Kenya: a cluster-randomized trial. Water and Health Conference: Where Science Meets Policy. Chapel Hill, NC.

2. Freeman M, Greene L, Dreibelbis R, Saboori S, Muga R, Brumback B, Rheingans R, 2011. Assessing the impact of a school-based water treatment, hygiene, and sanitation program on pupil absence in Nyanza Province, Kenya: A cluster-randomized trial. Tropical Medicine & International Health doi: 10.1111/j.1365-3156.2011.02927.x. [Epub ahead of print].

3. O'Reilly C, Freeman M, Ravani M, Migele J, A. M, Ayalo M, S. O, Hoekstra MR, E. QR, 2008. The impact of a school-based safe water and hygiene programme on knowledge and practices of students and their parents: Nyanza Province, western Kenya, 2006. Epidemiology & Infection 136: 80-91.

4. Dreibelbis R, Green L, Freeman M, Saboori S, Muga R, Rheingans R, 2011. Multi-level assessment of the association of primary school absence with school and household water, sanitation, and hygiene conditions: the influence of gender and household wealth. Submitted - Jounral of Education Development.

5. Freeman M, Clasen T, S S, Dreibelbis R, R R, 2011. Assessing the impact of a school-based water treatment, hygiene, and sanitation program onpupil diarrhea: A cluster-randomized trial. Faculty of Infectious Disease: London School of Hygiene and Tropical Medicine.

6. UNICEF, 2010. Raising Clean Hands: Advancing Learning, Health, and Participation through WASH in Schools.

7. Lieberman HR, 2007. Hydration and Cognition: A Critical Review and Recommendations for Future Research. Journal of the American College of Nutrition 26: 555S-561S.

8. Benton D, 2011. Dehydration Influences Mood and Cognition: A Plausible Hypothesis? Nutrients 3: 555-573.

9. Grandjean AC, Grandjean NR, 2007. Dehydration and Cognitive Performance. Journal of the American College of Nutrition 26: 549S-554S.

10. Bar-David Y, Urkin J, Kozminsky ELY, 2005. The effect of voluntary dehydration on cognitive functions of elementary school children. Acta Pædiatrica 94: 1667-1673.

11. P Bangirana JM, C John, M Boivin & J Hodges, 2013. The association between cognition and academic performance in ugandan children surviving malaria with neurological involvement. PLoS One 8.

12. Sawka MN, Cheuvront SN, Carter R, 2005. Human Water Needs. Nutrition Reviews 63: S30-S39.

13. Sharma VM, Sridharan K, Pichan G, Panwar MR, 1986. Influence of heat-stress induced dehydration on mental functions. Ergonomics 29: 791-799.

14. Gopinathan PM, Pichan G, Sharma VM, 1988. Role of Dehydration in Heat Stress-Induced Variations in Mental Performance. Archives of Environmental Health 43.

15. Baker LB, Conroy DE, Kenney WL, 2007. Dehydration Impairs Vigilance-Related Attention in Male Basketball Players. Medicine & Science in Sports & Exercise 39: 976-983.

16. Wilson MMG, Morley JE, 2003. Impaired cognitive function and mental performance in mild dehydration. European Journal of Clinical Nutrition 57: S24-S29.

17. Cian C, Barraud PA, Melin B, Raphel C, 2001. Effects of fluid ingestion on cognitive function after heat stress or exercise-induced dehydration. International Journal of Psychophysiology 42: 243-251.

18. Szinnai G, Schachinger H, Arnaud MJ, Linder L, Keller U, 2005. Effect of water deprivation on cognitive-motor performance in healthy men and women. American Journal of Physiology - Regulatory, Integrative and Comparative Physiology 289: R275-R280.

19. Armstrong LE, Ganio MS, Casa DJ, Lee EC, McDermott BP, Klau JF, Jimenez L, Le Bellego L, Chevillotte E, Lieberman HR, 2012. Mild Dehydration Affects Mood in Healthy Young Women. The Journal of Nutrition.

20. Susan M. Shirreffs, Stuart J. Merson, Fraser SM, Archer DT, 2004. The effects of fluid restriction on hydration status and subjective feelings in man. British Journal of Nutrition 91: 951-958

21. Ganio MS, Armstrong LE, Casa DJ, McDermott BP, Lee EC, Yamamoto LM, Marzano S, Lopez RM, Jimenez L, Bellego LL, Chevillotte E, Lieberman HR, 2011. Mild dehydration impairs cognitive performance and mood of men. Human and Clinical Nutrition 106: 1535-1543

22. Kempton MJ, Ettinger U, Foster R, Williams SCR, Calvert GA, Hampshire A, Zelaya FO, O'Gorman RL, McMorris T, Owen AM, Smith MS, 2011. Dehydration affects brain structure and function in healthy adolescents. Human Brain Mapping 32: 71-79.

23. Tomporowski PD, Beasman K, Ganio MS, Cureton K, 2007. Effects of Dehydration and Fluid Ingestion on Cognition. Int J Sports Med 28: 891,896.

24. Rogers PJ, Kainth A, Smit HJ, 2001. A drink of water can improve or impair mental performance depending on small differences in thirst. Appetite 36: 57-58.

25. Neave N, Scholey AB, Emmett JR, Moss M, Kennedy DO, Wesnes KA, 2001. Water ingestion improves subjective alertness, but has no effect on cognitive performance in dehydrated healthy young volunteers. Appetite 37: 255-256.

26. D'Anci KE, Constant F, Rosenberg IH, 2006. Hydration and Cognitive Function in Children. Nutrition Reviews 64: 457-464.

27. Bar-David Y, Urkin J, Landau D, Bar-David Z, Pilpel D, 2009. Voluntary dehydration among elementary school children residing in a hot arid environment. Journal of Human Nutrition and Dietetics 22: 455-460.

28. Benton D, Burgess N, 2009. The effect of the consumption of water on the memory and attention of children. Appetite 53: 143-146.

29. Edmonds CJ, Burford D, 2009. Should children drink more water?: The effects of drinking water on cognition in children. Appetite 52: 776-779.

30. Edmonds CJ, Jeffes B, 2009. Does having a drink help you think? 6–7-Year-old children show improvements in cognitive performance from baseline to test after having a drink of water. Appetite 53: 469-472.

31. Armstrong L, 2007. Journal of the American College of Nutrition. Assessing hydration status: the elusive gold standard 26: 575S–584S.

32. Kavouras S, 2002. Assessing hydration status. J Am Coll Nutr 26: 575S-584S.
